# ﻿Phylogeny and phenotype of *Filobasidium* revealing three new species (Filobasidiaceae, Filobasidiales) from China

**DOI:** 10.3897/mycokeys.114.142438

**Published:** 2025-02-24

**Authors:** Chun-Yue Chai, Zhi-Wen Xi, Qiu-Hong Niu, Feng-Li Hui

**Affiliations:** 1 School of Life Science, Nanyang Normal University, Nanyang 473061, China Nanyang Normal University Nanyang China; 2 Research Center of Henan Provincial Agricultural Biomass Resource Engineering and Technology, Nanyang Normal University, Nanyang 473061, China Nanyang Normal University Nanyang China

**Keywords:** Basidiomycetes, phylogenetic analysis, phylloplane, taxonomy

## Abstract

The genus *Filobasidium*, a member of the family Filobasidiaceae in the order Filobasidiales, is a group of basidiomycetes with many representative species. To date, 14 species have been described and accepted in *Filobasidium*. Although some newfound species from China have recently been published, the species diversity of *Filobasidium* remains incompletely understood. Samples from various areas of China were obtained and examined to investigate the species diversity of the genus. Three new species, namely *F.pseudomali***sp. nov.**, *F.castaneae***sp. nov.**, and *F.qingyuanense***sp. nov.**, were introduced based on phylogenetic analyses of the internal transcribed spacer (ITS) region and the D1/D2 domain of the large subunit (LSU) rRNA gene and the ITS sequence alone coupled with phenotypic characteristics. Full descriptions, illustrations, comparisons with similar species, and phylogenetic analyses are provided. Findings from this study substantially enrich the biodiversity of *Filobasidium* in China.

## ﻿Introduction

The genus *Filobasidium* was first characterized by Olive (1968) in the description of a sexual species, *F.floriforme*, resulting in the establishment of the Filobasidiaceae family. Four additional species, *F.capsuligenum* (Rodrigues de Miranda 1972), *F.uniguttulatum* (Kwon-Chung 1977), *F.elegans* (Bandoni et al. 1991), and *F.globisporum* (Bandoni et al. 1991), were later described according to phenotypic characteristics. Scorzetti et al. (2002) established a phylogeny of the genus *Filobasidium* based on the internal transcribed spacer (ITS) region and the D1/D2 domain of the large subunit (LSU) rRNA gene, and placed the genus in Filobasidiales. Five asexual *Cryptococcus* species, including *C.chernovii*, *C.magnus*, *C.oeirensis*, *C.stepposus*, and *C.wieringae*, are members of the *floriforme* clade as demonstrated by phylogenetic analyses of the small subunit (SSU), D1/D2 domain, and ITS region (Fell et al. 2000; Fonseca et al. 2000; Scorzetti et al. 2002; Golubev et al. 2006). According to the Melbourne Code (McNeill et al. 2012), which specifies that related anamorphic and teleomorphic species can be assigned to the same genus, these five asexual *Cryptococcus* species were consequently reassigned to the genus as the new combinations, *F.chernovii*, *F.magnum*, *F.oeirense*, *F.stepposum*, and *F.wieringae*, based on multi-gene phylogeny (Liu et al. 2015a, 2015b). Correspondingly, the unique fermentative species, *F.capsuligenum*, was removed from this genus as it was located outside the *Filobasidium* clade and was closely associated with the *Piskurozyma* clade (Liu et al. 2015b). In recent years, several new species, such as *F.dingjieense*, *F.globosum*, *F.mali*, *F.mucilaginum* (Li et al. 2020), and *F.chaidanensis* (Wei et al. 2022), have been published.

*Filobasidium* species can reproduce both asexually and sexually. Through asexual reproduction, the species reproduce by budding, and some species produce pseudohyphae and/or true hyphae with clamp connections and haustorial branches (Fonseca et al. 2011; Li et al. 2020; Wei et al. 2022). Alternatively, four sexual species, *F.floriforme*, *F.elegans*, *F.globisporum*, and *F.uniguttulatum*, generate long, slender, nonseptate basidia with terminal sessile basidiospores (Kwon-Chung 2011; Liu et al. 2015b). Most of the known *Filobasidium* species can grow on L-malic, saccharic, as well as protocatechuic and p-hydroxybenzoic acids, while nitrate utilization was observed in some species. The primary ubiquinone in the *Filobasidium* species is CoQ-9 or CoQ-10 (Liu et al. 2015b).

Members of the genus *Filobasidium* have been investigated for various biotechnological applications and pathology. Most previous studies have focused on the most widely distributed species, *F.magnum* (Wei et al. 2022). For instance, as a bio-transformer, the *F.magnum* strain JD1025 can effectively convert sclareol to sclareolide (Fang et al. 2023). Strain JD1025 of *F.magnum* can metabolize nobiletin for the biosynthesis of 6- and 7-mono-demethylated nobiletin (Su et al. 2022). Endophytic strains of *F.magnum* are associated with the formation of grape flavor, acting as a candidate for wine flavor enhancement (Sayed et al. 2021). Moreover, *F.globisporum* is frequently detected in industrial-scale malting processes. It can produce extracellular β-glucanase and cellulase with a potentially positive contribution to the malt enzyme spectrum (Laitila et al. 2006). *Filobasidiummagnus* and *F.unigutulatum* have been reported to exist as clinical specimens. However, only *F.magnus* can grow at the human body temperature, suggesting that it may be an opportunistic human pathogen (Fonseca et al. 2011; Aboutalebian et al. 2020; Baptista et al. 2020).

Currently, 14 species in the genus *Filobasidium* have been recorded in Mycobank [https://www.mycobank.org (accessed on 20 November 2024)]. In China, 13 *Filobasidium* species have been reported, encompassing six species initially described in China (Luo et al. 2019; Li et al. 2020; Wei et al. 2022). While some new species from China have recently been published, the diversity of *Filobasidium* remains only partially understood. In this study, seven basidiomycetous yeast strains were collected from Guangdong, Guizhou, and Henan Provinces of China. Morphological characteristics and phylogenetic analysis based on the combined ITS and LSU sequences and the ITS sequence alone revealed that these strains represent three undescribed species of *Filobasidium*. Our aim in this investigation is to employ an integrative taxonomic approach for the identification and description of these new taxa.

## ﻿Materials and methods

### ﻿Sample collection and yeast isolation

A total of 25 leaf samples were obtained from the Guangdong, Guizhou, and Henan Provinces of China. Leaf samples were stored in sterile plastic bags and kept in an icebox for 6–12 h during transfer to the laboratory. Yeast strains were isolated from leaf surfaces using the improved ballistospore-fall method, as described previously (Nakase and Takashima 1993). Vaseline was utilized to affix fresh and healthy leaves to the insides of Petri dishes filled with yeast extract-malt extract (YM) agar (0.3% yeast extract, 0.3% malt extract, 0.5% peptone, 1% glucose, and 2% agar). The YM agar plates were incubated at 20 °C until visible colonies formed. Different yeast morphotypes were chosen and purified by streaking on distinct YM agar plates. Following purification, yeast strains were suspended in 20% (v/v) glycerol and stored at −80 °C. Cultures for all obtained isolates were preserved in the Microbiology Lab at Nanyang Normal University, Henan, China. All isolates employed in this study and their origins are presented in Table 1.

**Table 1.** Yeast strains and isolation sources utilized in this study.

Strain	Source	Location	Date
*Filobasidiumpseudomali* sp. nov.			
NYNU 228108^T^	Leaf of *Photinia* sp.	Guiyang Medicinal Botanical Garden, Guiyang, Guizhou, China	August 2022
NYNU 22986	Leaf of *Litseacubeba*	Guiyang Medicinal Botanical Garden, Guiyang, Guizhou, China	August 2022
*Filobasidiumcastaneae* sp. nov.			
NYNU 2111105^T^	Leaf of *Castaneamollissima*	Baotianman Nature Reserve, Nanyang, Henan, China	November 2021
NYNU 23230	Leaf of *Mussaendapubescens*	Pingtang county, Buyi and Miao Autonomous Prefecture of Qian Nan, Guizhou, China	February 2023
NYNU 23245	Leaf of *Mussaendapubescens*	Pingtang county, Buyi and Miao Autonomous Prefecture of Qian Nan, Guizhou, China	February 2023
*Filobasidiumqingyuanense* sp. nov.			
NYNU 223211^T^	Leaf of *Lespedezaformosa*	Qingyuan Mountain, Quanzhou, Guangdong, China	March 2022
NYNU 23239	Leaf of *Mussaendapubescens*	Pingtang county, Buyi and Miao Autonomous Prefecture of Qian Nan, Guizhou, China	February 2023

### ﻿Phenotypic characterization

Morphological, physiological, and biochemical characteristics were assessed based on methods established by Kurtzman et al. (2011). Sexual processes of all strains were investigated on potato dextrose agar (PDA, 20% potato extract, 2% glucose, and 1.5% agar), corn meal (CM) agar (2.2% corn extract and 1.5% agar), and yeast carbon base supplemented with 0.01% ammonium sulfate (YCBS) agar at 17 °C for two months and observed weekly (Kwon-Chung 2011; Li et al. 2020). The inverted-plate method (do Carmo-Sousa and Phaff 1962) was employed to observe the ballistoconidium-forming activity of all yeasts following two weeks of incubation on CM agar at 17 °C. Glucose fermentation was performed in a liquid medium using Durham fermentation tubes. Carbon and nitrogen source assimilation tests were performed in a liquid medium, and starved inoculum was utilized for the nitrogen test (Kurtzman et al. 2011). Growth at different temperatures (15, 20, 25, 30, 35, and 37 °C) was characterized by growth on YM agar. Cell morphology was assessed using a Leica DM 2500 microscope (Leica Microsystems GmbH, Wetzlar, Germany) alongside a Leica DFC295 digital microscope color camera. All novel taxonomic descriptions and proposed names were deposited in the MycoBank database (http://www.mycobank.org).

### ﻿DNA extraction, PCR amplification, and sequencing

Genomic DNA was extracted from yeast strains using the Ezup Column Yeast Genomic DNA Purification Kit following the manufacturer’s instructions (Sangon Biotech Co., Shanghai, China). The ITS region, the D1/D2 domain of the LSU rRNA gene, the largest subunit of RNA polymerase I (*RPB1*) gene, and the second largest subunit of RNA polymerase II (*RPB2*) gene were amplified using primers ITS1/ITS4 (White et al. 1990), NL1/NL4 (Kurtzman and Robnett 1998), RPB1-Af/RPB1-Cr (Kurtzman and Robnett 2003), and RPB2-5F/RPB2-7cAR (Kurtzman and Robnett 2003), respectively. Amplification was performed in a 25 µL reaction-volume tube containing 9.5 µL of ddH_2_O, 12.5 µL of 2 × Taq PCR Master Mix with blue dye (Sangon Biotech Co., Shanghai, China), 1 µL of DNA template, and 1 µL of each primer. PCR was conducted as described by Toome et al. (2013) for the ITS and LSU regions. For the partial *RPB1* and *RPB2* genes, we utilized a touchdown PCR protocol as previously described (Wang et al. 2014). The PCR products were purified and sequenced at Sangon Biotech Co., Ltd (Shanghai, China) using the same primers. We determined the identity and accuracy of the newly obtained sequences by comparing them to sequences found in the GenBank database and assembled them using BioEdit v. 7.1.3.0 (Hall 1999). All newly generated sequences were deposited in the GenBank database (https://www.ncbi.nlm.nih.gov/genbank/).

### ﻿Phylogenetic analysis

Sequences generated in this study and those obtained from GenBank (Table 2) were used for phylogenetic analyses. Firstly, the combined dataset of the ITS and LSU regions was used to explore the phylogenetic positions of the newly studied specimens within *Filobasidium*. Secondly, the ITS sequence alone was used to further differentiate species identities within this genus. Each dataset was aligned using MAFFT v. 7.110 (Katoh and Standley 2013) with the G-INI-I option. Alignments were visualized, trimmed, and edited, where necessary, using MEGA v.7.0.26 (Kumar et al. 2016). Regarding the combined dataset of the ITS and LSU regions, each region was aligned separately, and then the alignments of the two regions were concatenated as a single alignment.

**Table 2.** Taxa included in molecular phylogenetic analyses and their GenBank accession numbers. Entries in bold were newly generated for this study.

Taxa name	Sample	GenBank accession numbers	
		ITS	LSU D1/D2
***Filobasidiumcastaneae* sp. nov.**	**NYNU 2111105^T^**	**OM049430**	**OM049431**
***Filobasidiumcastaneae* sp. nov.**	**NYNU 23230**	**PP114094**	**PP114092**
***Filobasidiumcastaneae* sp. nov.**	**NYNU 23245**	**PP114096**	**PP114097**
*Filobasidiumchaidanensis*	CGMCC 2.6796^T^	OM417191	OM417191
*Filobasidiumchernovii*	CBS 8679^T^	NR_073223	NG_068965
*Filobasidiumdingjieense*	CGMCC 2.5649^T^	NR_174759	MK050342
*Filobasidiumelegans*	CBS 7640^T^	AF190006	AF181548
*Filobasidiumfloriforme*	CBS 6241^T^	NR_119429	NG_069409
*Filobasidiumglobisporum*	CBS 7642^T^	NR_119453	NG_070553
*Filobasidiumglobosum*	CGMCC 2.5680^T^	NR_174760	MK050344
*Filobasidiummagnum*	CBS 140^T^	NR_130655	NG_069409
*Filobasidiummali*	CGMCC 2.4012^T^	NR_174761	MK050346
*Filobasidiummucilaginum*	CGMCC 2.3463^T^	NR_174762	MK050349
*Filobasidiumoeirensis*	CBS 8681^T^	NR_077106	NG_070508
***Filobasidiumpseudomali* sp. nov.**	**NYNU 228108^T^**	**OP581930**	**OP566876**
***Filobasidiumpseudomali* sp. nov.**	**NYNU 22986**	**PP108743**	**PP108744**
***Filobasidiumqingyuanense* sp. nov.**	**NYNU 223211^T^**	**OP278683**	**OP278680**
***Filobasidiumqingyuanense* sp. nov.**	**NYNU 23239**	**PP114093**	**PP114095**
*Filobasidiumstepposum*	CBS 10265^T^	NR_111207	KY107724
*Filobasidiumuniguttulatum*	CBS 1730^T^	NR_111070	NG_056269
*Filobasidiumwieringae*	CBS 1937^T^	NR_077105	NG_067314
*Filobasidium* sp.	KBP Y-5548	MH697755	MH697755
*Filobasidium* sp.	UFMG-CM-Y6635	OM480729	OM321340
‘*Cryptococcus*’ sp.	2 IA06	KM246189	KM246106
‘*Cryptococcus*’ sp.	2 MG34	KM246229	KM246145
‘*Cryptococcus*’ sp.	11-1115	KM986117	KM206723
‘*Cryptococcus*’ sp.	RP419_8	KX067803	KX067803
*Goffeauzymaaciditolerans*	CBS 10872^T^	NR_137808	NG_058295
*Goffeauzymagastrica*	CBS 2288^T^	NR_111048	NG_058296
Uncultured fungus clone	OTU_812	MH365273	–
Uncultured fungus clone	–	LR880016	–
Uncultured fungus clone	–	LR136377	–
Uncultured fungus clone	–	LT995797	–

CBS, CBS-KNAW Collections, Westerdijk Fungal Biodiversity Institute, Utrecht, The Netherlands; CGMCC, China General Microbiological Culture Collection Center, Beijing, China; NYNU, Microbiology Lab, Nanyang Normal University, Henan, China; **^T,^** type strain.

Maximum likelihood (ML) and Bayesian inference (BI) methods were utilized for phylogenetic analyses. The ML method was conducted with RAxML v. 8.2.3 using the GTRGAMMA model (Stamatakis 2014). ML bootstrap values (MLBS) were evaluated using 1,000 rapid bootstrap replicates. For the BI approach, the optimal evolutionary model for each partition was determined using ModelFinder (Kalyaanamoorthy et al. 2017). The BI method was performed with MrBayes v. 3.2.7a (Ronquist et al. 2012) in the CIPRES Science Gateway version 3.3. Six simultaneous Markov chains were performed over 50 million generations, and trees were sampled every 1,000 generation. The first 25% of the created sample trees were removed as they represent the burn-in phase of analysis. The remaining trees were employed to determine the Bayesian posterior probabilities (BPP). FigTree v. 1.4.3 was used to visualize the phylogenetic trees (Andrew 2016). Branches with bootstrap values for MLBS ≥ 50% and BPP ≥ 0.95 were considered significantly supported.

## ﻿Results

### ﻿Yeast isolation and diversity

During this study, 106 yeast strains were isolated from 25 leaf samples collected in the Guangdong, Guizhou, and Henan Provinces of China. All strains were identified to the species level based on the threshold of >99% sequence identity with the type strain of a described species in the D1/D2 domain or ITS region (Kurtzman and Robnett 1998; Fell et al. 2000; Scorzetti et al. 2002; Vu et al. 2016). A total of 95 strains present in the samples were classified as Basidiomycota belonging to 20 species in 12 genera: *Bannoaogasawarensis*, *Bulleraalba*, *Bulleramrakii*, *Bulleribasidiumpseudovariabile*, *Cystobasidiumpallidum*, *Derxomyceskomagatae*, *Dioszegiahungarica*, *Erythrobasidiumhasegawianum*, *Hannaellasinensis*, *Hannaellataiwanensis*, *Sporidiobolusmetaroseus*, *Sporobolomycescarnicolor*, *Sporobolomycesroseus*, *Tilletiopsiswashingtonensis*, *Vishniacozymafoliicola*, *Vishniacozymacarnescens*, *Vishniacozymavictoriae*, and three *Filobasidium* species that are not yet formally described and therefore represent new species. In addition, eleven strains belonging to Ascomycota were also obtained from these samples. The ascomycetous yeasts were found to be four known species in four genera: *Aureobasidiumpullulans*, *Candidasilvanorum*, *Yamadazymascolyti*, and *Wickerhamomycessydowiorum*. Among the 24 species identified, *Tilletiopsiswashingtonensis* was the most dominant species, which occurred in six samples collected from different locations, while *Bannoaogasawarensis*, *Bulleramrakii*, and *Yamadamycesterricola* occurred only in one sample or location.

### ﻿Phylogeny of novel yeast species

Seven specimens preliminarily identified as *Filobasidium* were studied further. ITS and LSU regions were newly generated from all these specimens (Table 2).

**Figure 1.**
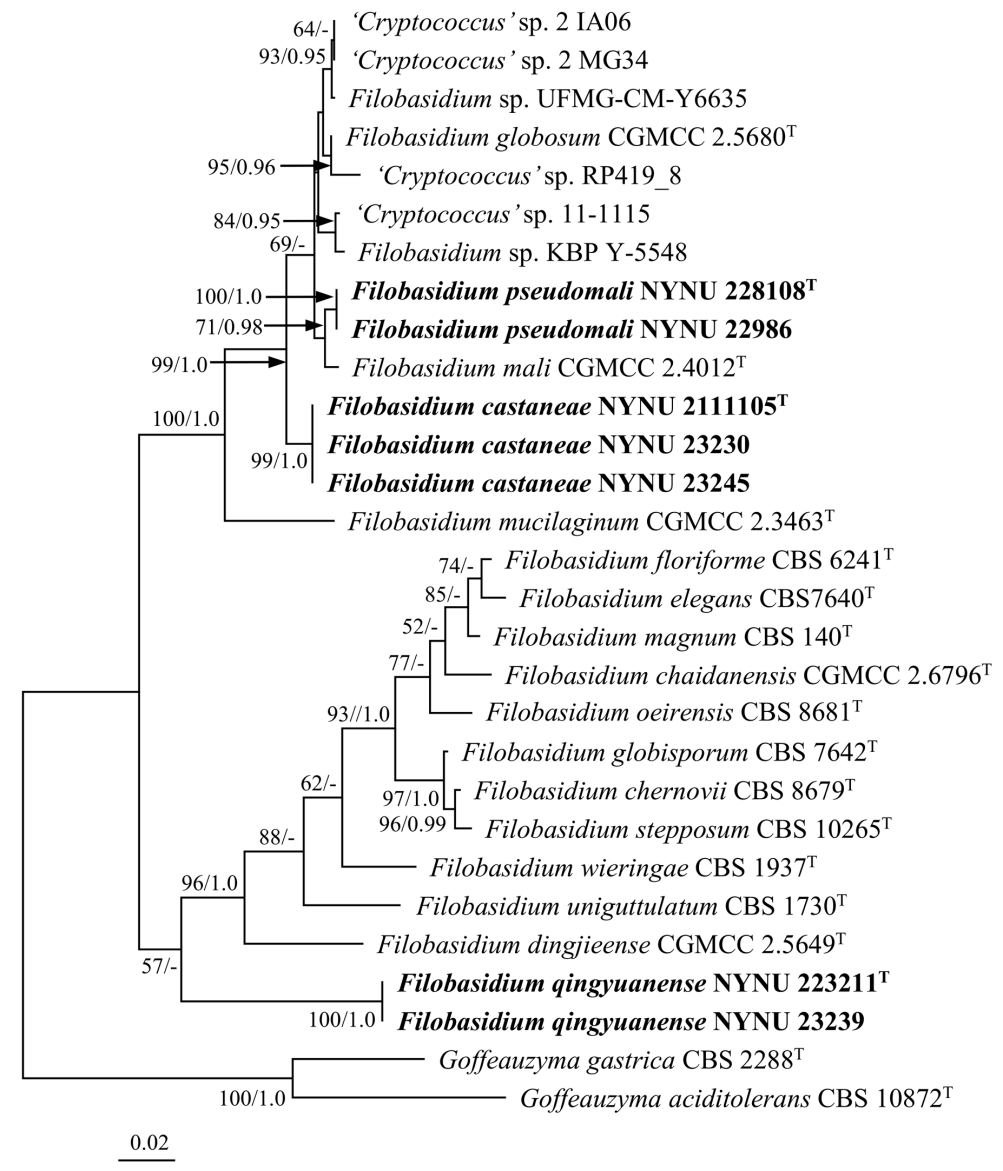
Maximum likelihood (ML) phylogenetic tree of *Filobasidium* derived from combined ITS and LSU sequence data. The tree is rooted with *Goffeauzymagastrica*CBS 2288^T^ and *Goffeauzymaaciditolerans*CBS 10872^T^. Bootstrap values (MLBS ≥ 50% and BPP ≥ 0.95) are shown around branches. Sequences from type strains are marked with (T), and the new species are indicated in bold.

The combined dataset of ITS and LSU regions consisted of 29 sequences from 24 taxa, including 14 newly generated sequences (seven for ITS and seven for LSU). The final alignment included 1,124 characters (486 characters from ITS and 637 characters from LSU), of which 752 were constant, 372 were variable, 279 were parsimony-informative, and 93 were singletons. Both ML and BI methods produced similar topologies in the main lineages. The ML-derived topology, along with MLBS and BPP values above 50% and 0.95, respectively, is presented (Fig. 1). The phylogeny indicated that seven strains isolated in this study formed three highly supported groups (Fig. 1) within the genus and were distinct from other species of *Filobasidium*.

**Figure 2.**
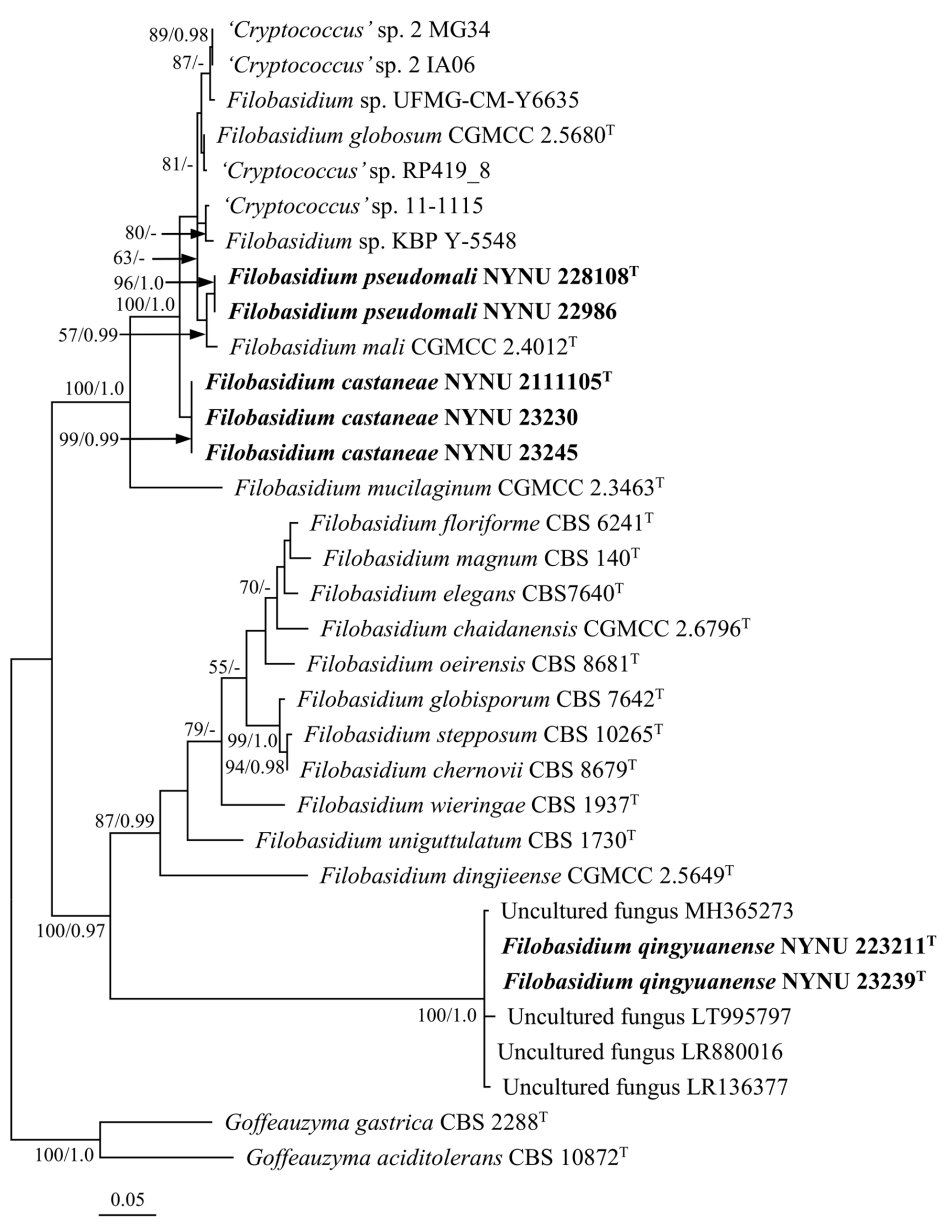
Maximum likelihood (ML) phylogenetic tree of *Filobasidium* derived from ITS sequence data. The tree is rooted with *Goffeauzymagastrica*CBS 2288^T^ and *Goffeauzymaaciditolerans*CBS 10872^T^. Bootstrap values (MLBS ≥ 50% and BPP ≥ 0.95) are shown around branches. Sequences from type strains are marked with (T), and the new species are indicated in bold.

The ITS dataset consisted of 33 sequences from 24 taxa, including seven newly generated sequences. The final alignment included 486 characters, of which 267 were constant, 219 were variable, 195 were parsimony-informative, and 26 were singletons. The ML and BI methods yielded similar topologies in the main lineages. The ML-derived topology, with MLBS and BPP values above 50% and 0.95, respectively, is shown (Fig. 2). This tree demonstrated 14 known *Filobasidium* species, while the newly isolated strains formed three independent groups, consistent with the combined ITS and LSU dataset phylogeny.

Strains NYNU 228108 and NYNU 22986 had identical sequences in the D1/D2 domain and ITS region, indicating their conspecificity. In the phylogenetic tree, two strains formed a well-supported clade grouped with *F.mali* with moderate support (Figs 1, 2). They differed from their closest relative, *F.mali*, by two nucleotide (nt) substitutions in the D1/D2 domain. However, they differed by 15 nt (~2.5%) mismatches from *F.mali* in the ITS region. Generally, the basidiomycetous yeast strains differing by two or more nucleotide substitutions in the D1/D2 domain or 1–2% nucleotide differences in the ITS region may represent different taxa (Scorzetti et al. 2002). Recently, Li et al. (2020) determined the number of nucleotide variations and sequence similarities in the D1/D2 domain and ITS region among the type strains of species from 40 genera of *Agaricomycotina* and 30 genera of *Pucciniomycotina*. They demonstrated that the nucleotide variation among the strains of *Filobasidium* species is 0–21 nt (~0–3.3%) in the D1/D2 domain and 4–106 nt (~0.7–5.8%) in the ITS region. The sequence divergences in the D1/D2 and ITS regions have raised the possibility that the two strains may represent a novel species distinct from *F.mali*. Moreover, the partial *RPB1* and *RPB2* gene sequences further confirmed the novelty of this species, as the two strains differed by 114 (~16.8%) and 143 (~12.9%) nt substitutions from *F.mali* in these regions. The sequence comparison and results of the phylogenetic analyses indicated that the strains NYNU 228108 and NYNU 22986 represent a novel species of *Filobasidium*. Therefore, the name *Filobasidiumpseudomali* sp. nov. is proposed to accommodate these two strains.

Strains NYNU 2111105, NYNU 23230, and NYNU 23245 with identical sequences in the D1/D2 domain and ITS region formed a separate clade, clustering alongside *F.globosum*, *F.mali*, *F.pseudomali* sp. nov., and five unpublished strains, *Filobasidium* sp. KBP Y-5548, *Filobasidium* sp. UFMG-CM-Y6635, ‘*Cryptococcus*’ sp. RP419_8, ‘*Cryptococcus*’ sp. 2 IA06, and ‘*Cryptococcus*’ sp. 2 MG34, with high support (Figs 1, 2). They differed from the above three described species and five unpublished strains by 5–7 nt (~0.8–1.2%) substitutions in the D1/D2 domain and by more than 23 nt (~3.9%) mismatches in the ITS region. Thus, these three strains represent a novel *Filobasidium* species, for which the name *Filobasidiumcastaneae* sp. nov. is proposed.

Strains NYNU 223211 and NYNU 23239 possessed identical sequences in the D1/D2 domain and ITS region, forming a subclade with four uncultured fungus clones (MH365273, LR880016, LR136377, and LT995797) in the tree of the ITS dataset (Fig. 2). A BLASTn search of the ITS sequences revealed that NYNU 223211 and NYNU 23239 had 99.2–100% sequence similarities with four uncultured fungus clones, which indicated that they may be conspecific. In the tree of the combined ITS and LSU dataset, strains NYNU 223211 and NYNU 23239 formed separate branches at the bottom of the *Filobasidium* clade (Fig. 1). They differed from other known *Filobasidium* species by 18 nt (~3%) substitutions in the D1/D2 domain and more than 34 nt (~9.3%) mismatches in the ITS region, suggesting that they represent a novel *Filobasidium* species. Therefore, a novel species, *Filobasidiumqingyuanense*, is proposed to accommodate these two strains.

### ﻿Taxonomy

#### ﻿ Filobasidium pseudomali

Taxon classificationFungiFilobasidialesFilobasidiaceae

C.Y. Cai & F.L. Hui sp. nov.

86625911-4C54-5F43-BD7F-9168C70758AE

851823

Fig. 3A

##### Etymology.

The specific epithet *pseudomali* refers to similar colony morphological and physiological characteristics to that of *Filobasidium mali*.

##### Typus.

China • Guizhou Province, Guiyang City, Guiyang Medicinal Botanical Garden, in the phylloplane of *Photinia* sp., August 2022, L. Zhang and F.L. Hui, NYNU 228108 (holotype GDMCC 2.305^T^ preserved in a metabolically inactive state in Guangdong Microbial Culture Collection Center, culture ex-type PYCC 9928 deposited in the Portuguese Yeast Culture Collection).

##### Description.

On YM agar, after 7 days at 20 °C, the streak culture is gray-cream, mucoid, smooth, and glossy. The margin is entire. On YM agar, after 7 days at 20 °C, cells are globosal and ellipsoidal, 3.8–6.4 × 5.2–8.4 μm, and single, budding is polar. After 1 month at 20 °C, a ring and sediment are present. In Dalmau plate culture on corn meal agar, pseudohyphae are not formed. Sexual structures are not observed on PDA, CM agar, and YCBS agar for two months. Ballistoconidia are not produced. Glucose fermentation is absent. Glucose, inulin, sucrose, raffinose, melibiose, galactose, lactose, trehalose, maltose, melezitose, methyl-α-D-glucoside, cellobiose, L-sorbose, L-rhamnose, D-xylose, L-arabinose, D-arabinose, 5-keto-D-gluconate, ethanol, ribitol, galactitol, D-mannitol, D-glucitol, myo-inositol, succinate, citrate, D-gluconate, 2-keto-D-gluconate, D-glucuronate, and glucono-1,5-lactone are assimilated as sole carbon sources. Salicin, D-ribose, methanol, glycerol, erythritol, DL-lactate, D-glucosamine, and N-acetyl-D-glucosamine are not assimilated. Nitrate, nitrite, ethylamine, and L-lysine (weak) are assimilated as sole nitrogen sources. Cadaverine is not assimilated. Maximum growth temperature is 30 °C. Growth in vitamin-free medium is positive. Growth on 50% (w/w) glucose-yeast extract agar is negative. Starch-like substances are not produced. Urease activity is positive. Diazonium Blue B reaction is positive.

**Figure 3.**
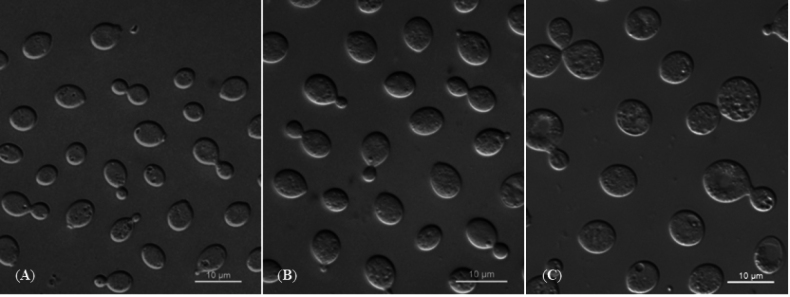
Vegetative cells of *F.pseudomali* sp. nov. NYNU 228108^T^ (**A**), *F.castaneae* sp. nov. NYNU 2111105^T^ (**B**), and *F.qingyuanense* sp. nov. NYNU 223211^T^ (**C**) following growth in YM broth for 7 days at 20 °C. Scale bars: 10 μm.

##### Additional strain examined.

China • Guizhou Province, Guiyang City, Guiyang Medicinal Botanical Garden, in the phylloplane of *Litseacubeba*, August 2022, L. Zhang and F.L. Hui, NYNU 22986.

##### GenBank accession numbers.

Holotype GDMCC 2.305^T^ (ITS: OP581930, D1/D2: OP566876, *RBP1*: OR963293, *RBP2*: PP151258); additional strain NYNU 22986 (ITS: PP108743, D1/D2: PP108744, RBP1: PP841943, RBP2: PP151259).

##### Note.

*Filobasidiumpseudomali* sp. nov. can be physiologically distinguished from its closest known species, *F.mali*, by its ability to assimilate inulin and citrate and its inability to assimilate salicin and cadaverine. Additionally, *F.pseudomali* nov. can grow in a vitamin-free medium, while *F.mali* cannot (Table 3).

**Table 3.** Physiological and biochemical features differing between the new species and closely related species.

Characteristics	1	2*	3*	4	5	6*
Carbon assimilation						
Inulin	+	–	+	+	+	–
Salicin	–	+/w	–	+	+	–
L-Sorbose	+	+	–	+	–	–
L-Rhamnose	+	+/d/w	d/w	+	+	–
D-Arabinose	+	v	–	-	+	–
D-Ribose	–	–	–	+	–	–
Glycerol	–	–	–	+	w	d/w
Ethanol	+	+/w	–	–	–	d/w
Ribitol	+	+	–	+	+	–
Galactitol	+	+	–	+	–	–
D-Mannitol	+	+	+	+	+	–
D-Glucitol	+	v	–	+	+	–
Citrate	+	–	–	+	+	+
Nitrogen assimilation						
Nitrite	+	–	–	+	+	+
Cadaverine	–	+	+	–	–	d/w
L-Lysine	d	–	+	n	n	n
Growth tests						
Growth in vitamin-free medium	+	–	+	+	+	n
Growth at 25 °C	+	+	+	+	+	–
Growth at 30 °C	+	+	–	–	+	–

1, *F.pseudomali* sp. nov.; 2, *F.mali*; 3, *F.globosum*; 4, *F.castaneae* sp. nov.; 5, *F.qingyuanense* sp. nov.; 6, *F.dingjieense*; +, positive reaction; –, negative reaction; d, delayed positive; w, weakly positive; n, data not available. All data from this study, except * which were obtained from the original description (Li et al. 2020).

#### ﻿ Filobasidium castaneae

Taxon classificationFungiFilobasidialesFilobasidiaceae

C.Y. Cai & F.L. Hui sp. nov.

54A3FD96-71DC-5F21-8325-6E7B14AE499D

851825

Fig. 3B

##### Etymology.

The specific epithet *castaneae* refers to *Castanea*, the plant genus from which the type strain was isolated.

##### Typus.

China • Henan Province, Nanyang City, Baotianman Nature Reserve, in the phylloplane of *Castaneamollissima*, November 2021, R.R. Jia and W.T. Hu, NYNU 2111105 (holotype CICC 33541^T^ preserved in a metabolically inactive state in the China Centre of Industrial Culture Collection, culture ex-type JCM 35729 deposited in the Japan Collection of Microorganisms).

##### Description.

On YM agar, after 7 days at 20 °C, the streak culture is gray-cream, mucoid, smooth, and glossy. The margin is entire. On YM agar, after 7 days at 20 °C, cells are globosal and ellipsoidal, 3.6–5.8 × 4.2–7.1 μm, and single, budding is polar. After 1 month at 20 °C, a ring and sediment are present. In Dalmau plate culture on corn meal agar, pseudohyphae are not formed. Sexual structures are not observed on PDA, CM agar, and YCBS agar for two months. Ballistoconidia are not produced. Glucose fermentation is absent. Glucose, inulin, sucrose, raffinose, melibiose, galactose, lactose, trehalose, maltose, melezitose, methyl-α-D-glucoside, cellobiose, salicin, L-sorbose, L-rhamnose, D-xylose, L-arabinose, 5-keto-D-gluconate, D-ribose, glycerol, ribitol, galactitol, D-mannitol, D-glucitol, myo-inositol, succinate, citrate, D-gluconate, N-acetyl-D-glucosamine, 2-keto-D-gluconate, D-glucuronate, and glucono-1,5-lactone are assimilated as sole carbon sources. D-Arabinose, methanol, ethanol, erythritol, DL-lactate, and D-glucosamine are not assimilated. Nitrate, nitrite, ethylamine, and L-lysine are assimilated as sole nitrogen sources. Cadaverine is not assimilated. Maximum growth temperature is 25 °C. Growth in vitamin-free medium is positive. Growth on 50% (w/w) glucose-yeast extract agar is negative. Starch-like substances are not produced. Urease activity is positive. Diazonium Blue B reaction is positive.

##### Additional strain examined.

China • Guizhou Province, Buyi and Miao Autonomous Prefecture of Qian Nan, Pingtang County, in the phylloplane of *Mussaendapubescens*, February 2023, D. Lu, NYNU 23230 and NYNU 23245.

##### GenBank accession numbers.

Holotype CICC 33541^T^ (ITS: OM049430, D1/D2: OM049431); additional strains NYNU 23230 (ITS: PP114094, D1/D2: PP114092) and NYNU 23245 (ITS: PP114096, D1/D2: PP114097).

##### Note.

*Filobasidiumcastaneae* sp. nov. can be physiologically distinguished from its closely related species *F.globosum*, *F.mali*, and *F.pseudomali* sp. nov. through its ability to assimilate D-ribose and glycerol (Table 3).

#### ﻿ Filobasidium qingyuanense

Taxon classificationFungiFilobasidialesFilobasidiaceae

C.Y. Cai & F.L. Hui sp. nov.

14191474-21D1-549D-B7A2-C29520BFC41F

851824

Fig. 3C

##### Etymology.

The specific epithet *qingyuanense* refers to the geographic origin of the type strain, Qingyuan Mountain, Quanzhou City, Guangdong Province.

##### Typus.

China • Guangdong Province, Quanzhou City, Qingyuan Mountain, in the phylloplane of *Lespedezaformosa*, March 2022, W.T. Hu and S.B. Chu, NYNU 223211 (holotype GDMCC 2.309^T^ preserved as a metabolically inactive state in the Guangdong Microbial Culture Collection Center, culture ex-type PYCC 9927 deposited in the Portuguese Yeast Culture Collection).

##### Description.

On YM agar, after 7 days at 20 °C, the streak culture is gray-cream, mucoid, smooth, and glossy. The margin is entire. On YM agar, after 7 days at 20 °C, cells are globosal and ellipsoidal, 6.7–10.2 × 7.6–10.4 μm and single, budding is polar. After 1 month at 20 °C, a ring and sediment are present. In Dalmau plate culture on corn meal agar, pseudohyphae are not formed. Sexual structures are not observed on PDA, CM agar, and YCBS agar for two months. Ballistoconidia are not produced. Glucose fermentation is absent. Glucose, inulin, sucrose, raffinose, melibiose, galactose, lactose, trehalose, maltose, melezitose, methyl-α-D-glucoside, cellobiose, salicin, L-rhamnose, D-xylose, L-arabinose, D-arabinose, 5-keto-D-gluconate, glycerol (weak), ribitol, D-mannitol, D-glucitol, myo-inositol, succinate, citrate, D-gluconate, 2-keto-D-gluconate, D-glucuronate, and glucono-1,5-lactone are assimilated as sole carbon sources. L-Sorbose, D-ribose, methanol, ethanol, erythritol, galactitol, DL-lactate, D-glucosamine, and N-acetyl-D-glucosamine are not assimilated. Nitrate, nitrite, ethylamine, and L-lysine are assimilated as sole nitrogen sources. Cadaverine is not assimilated. Maximum growth temperature is 30 °C. Growth in vitamin-free medium is positive. Growth on 50% (w/w) glucose-yeast extract agar is negative. Starch-like substances are not produced. Urease activity is positive. Diazonium Blue B reaction is positive.

##### Additional strain examined.

China • Guizhou Province, Qianxinan Buyei and Miao Autonomous Prefecture, Pingtang County, in the phylloplane of *Mussaendapubescens*, February 2023, D. Lu, NYNU 23239.

##### GenBank accession numbers.

Holotype GDMCC 2.309^T^ (ITS: OP278683, D1/D2: OP278680); additional strain NYNU 23239 (ITS: PP114093, D1/D2: PP114095).

##### Note.

*Filobasidiumqingyuanense* sp. nov. can be physiologically distinguished from its closest known species, *F.dingjieense*, by its ability to assimilate inulin, raffinose, melibiose, lactose, salicin, L-rhamnose, ribitol, D-mannitol, and D-glucitol, as well as an inability to assimilate ethanol. Additionally, *F.qingyuanense* nov. can grow at 25 °C, while *F.dingjieense* cannot (Table 3).

## ﻿Discussion

The present study described three new species (*F.pseudomali* sp. nov., *F.castaneae* sp. nov., and *F.qingyuanense* sp. nov.) based on phylogenetic analyses and phenotypic characteristics. Phylogenetically, these three species fell within the *Filobasidium* clade and were separated from other known species of *Filobasidium* and each other (Figs 1, 2). In contrast, phenotypically, all three species possessed similar cell shape, colony morphology, and color, differing from the closest known species in physiological and biochemical characteristics (Table 3). Phylogenetic analyses and phenotypic characteristics documented in this study confirm the existence of these new species in China.

Since the inception of *Filobasidium* in 1968, several *Filobasidium* species have been described based on phenotype (Kwon-Chung 1977; Bandoni et al. 1991). The classification based on phenotypical features, however, was in many cases not consistent with the results obtained from phylogenetic analyses. With the development of molecular biology, ribosomal DNA gene sequencing technology has been widely employed for yeast identification. The D1/D2 domain of the LSU rRNA gene is the most commonly used molecular marker for species delimitation of *Filobasidium* through phylogenetic analysis, as revealed by Scorzetti et al. (2002) and Kwon-Chung (2011). However, strains of different *Filobasidium* species sometimes shared identical or similar D1/D2 sequences but showed distinct sequences of the ITS region (Fell et al. 2000). Scorzetti et al. (2002) suggested that both gene regions are necessary for reliable species delimitation. For example, zero to two substitutions are present in the D1/D2 domain of the ex-type strains of the closest related species within *Filobasidium*, including *F.floriforme* and *F.magnum* (zero nt difference), *F.globosum* and *F.mali* (one nt difference), and *F.floriforme* and *F.oeirense* (two nt differences) (Li et al. 2020). Likewise, *F.pseudomali*, described in this study, differed from its close relative *F.mali* by only two nt substitutions. The other gene markers, including *RPB1*, *RPB2*, and the translation elongation factor 1-alpha (*TEF1*), exhibit increased variation between these closely related, well-defined species relative to the low nucleotide differences in the D1/D2 domain (Liu et al. 2018; Li et al. 2020). Although the D1/D2 domain is still an appropriate marker to use for higher-level taxon delimitations, it is clear that this region alone is insufficient for all species delimitation in the *Filobasidium*. Therefore, the data obtained from multiple genetic markers can allow for more accurate insights into the relationships between distinct taxa within *Filobasidium*.

Members of the genus *Filobasidium* have been found in diverse substrates, especially plant materials, including flowers, leaves, and fruit. More than 50% of the described *Filobasidium* species are associated with plant materials (Olive 1968; Kwon-Chung 1977; Bandoni et al. 1991; Kemler et al. 2017; Li et al. 2020; Wei et al. 2022). Strains of *Filobasidium* species have also been isolated from soil (Bandoni et al. 1991; Hong et al. 2002; Vishniac 2006; Fonseca et al. 2011; Yurkov 2017; Yurkov 2018; Li et al. 2020; Wei et al. 2022) and glacier ice (Fonseca et al. 2011). In addition, *F.globisporum* has been recognized as a relevant yeast species for the malting processes (Laitila et al. 2006). Furthermore, several phylloplane isolates of *F.magnus* and *F.wieringae* had multiple enzymatic activities, specifically the capacity to hydrolyze gelatin, casein, carboxymethyl-cellulose, and polygalacturonic acid to varying degrees (Fonseca et al. 2011). The biotechnological relevance of these hydrolytic activities has not been assessed, but they may have ecological relevance in the decomposition of plant material. In this study, we isolated seven strains of three new *Filobasidium* species, *F.pseudomali* sp. nov., *F.castaneae* sp. nov., and *F.qingyuanense* sp. nov., in the phylloplane, which may have similar ecological roles as *F.magnus* and *F.wieringae*.

## Supplementary Material

XML Treatment for Filobasidium pseudomali

XML Treatment for Filobasidium castaneae

XML Treatment for Filobasidium qingyuanense

## ﻿Additional information

### Author ORCIDs

Chun-Yue Chai https://orcid.org/0000-0003-0284-5560

Zhi-Wen Xi https://orcid.org/0000-0002-5814-5283

Qiu-Hong Niu https://orcid.org/0000-0003-1695-7117

Feng-Li Hui https://orcid.org/0000-0001-7928-3055

### Data availability

All of the data that support the findings of this study are available in the main text.
